# A Commercial Clay-Based Material as a Carrier for Targeted Lysozyme Delivery in Animal Feed

**DOI:** 10.3390/nano13222965

**Published:** 2023-11-17

**Authors:** Marianna Guagliano, Cinzia Cristiani, Matteo Dell’Anno, Giovanni Dotelli, Elisabetta Finocchio, Maria Lacalamita, Ernesto Mesto, Serena Reggi, Luciana Rossi, Emanuela Schingaro

**Affiliations:** 1 Dipartimento di Chimica, Materiali e Ingegneria Chimica “Giulio Natta”, Politecnico di Milano, Piazza Leonardo Da Vinci 32, 20133 Milano, Italy; giovanni.dotelli@polimi.it; 2 Dipartimento di Medicina Veterinaria e Scienze Animali—DIVAS, Università degli Studi di Milano, Via dell’Università 6, 26900 Lodi, Italy; matteo.dellanno@unimi.it (M.D.); serena.reggi@unimi.it (S.R.); luciana.rossi@unimi.it (L.R.); 3 Dipartimento di Ingegneria Civile, Chimica e Ambientale, Università di Genova, Via Opera Pia 15, 16145 Genova, Italy; elisabetta.finocchio@unige.it; 4 Dipartimento di Scienze della Terra e Geoambientali, Università degli Studi di Bari Aldo Moro, Via Edoardo Orabona 4, 70125 Bari, Italy; maria.lacalamita@uniba.it (M.L.); ernesto.mesto@uniba.it (E.M.); emanuela.schingaro@uniba.it (E.S.)

**Keywords:** clay-based materials, lysozyme–carrier interactions mechanism, target release, feed application, FT-IR spectroscopy, target delivery, precision nutrition

## Abstract

The controlled supply of bioactive molecules is a subject of debate in animal nutrition. The release of bioactive molecules in the target organ, in this case the intestine, results in improved feed, as well as having a lower environmental impact. However, the degradation of bioactive molecules’ in transit in the gastrointestinal passage is still an unresolved issue. This paper discusses the feasibility of a simple and cost-effective procedure to bypass the degradation problem. A solid/liquid adsorption procedure was applied, and the operating parameters (pH, reaction time, and LY initial concentration) were studied. Lysozyme is used in this work as a representative bioactive molecule, while Adsorbo^®^, a commercial mixture of clay minerals and zeolites which meets current feed regulations, is used as the carrier. A maximum LY loading of 32 mg_LY_/g_AD_ (LY(32)-AD) was obtained, with fixing pH in the range 7.5–8, initial LY content at 37.5 mg_LY_/g_AD_, and reaction time at 30 min. A full characterisation of the hybrid organoclay highlighted that LY molecules were homogeneously spread on the carrier’s surface, where the LY–carrier interaction was mainly due to charge interaction. Preliminary release tests performed on the LY(32)-AD synthesised sample showed a higher releasing capacity, raising the pH from 3 to 7. In addition, a preliminary Trolox equivalent antioxidant capacity (TEAC) assay showed an antioxidant capacity for the LY of 1.47 ± 0.18 µmol TroloxEq/g with an inhibition percentage of 33.20 ± 3.94%.

## 1. Introduction

Nutrition has become increasingly important, and four out of the seventeen sustainability goals (SDG 2 Zero Hunger, SDG 3 Good Health and Well-being, SDG6 Clean Water and Sanitation, SDG12 Responsible Consumption and Production, and SDG13 Climate Action) are targeted at the production of healthy food. In livestock, the increasing problem of antimicrobial resistance has prompted the European Union to introduce new limitations regarding the judicious use of antimicrobial molecules [1,2].

The indiscriminate use of not only antibiotics but also bioactive molecules in feed applications has a strong impact on the environment. Unmetabolized molecules, or parts of them when in excess, are released in both aquatic and atmospheric environments, generating issues of high levels of pollution [3]. Regarding antimicrobial activities, the use of functional feed additives [4,5,6,7] reduces the incidence of pathologies, and thus the amount of antibiotics needed [8,9,10,11,12]. Among other molecules [13], lysozyme (LY), a lytic enzyme [14] that exhibits antibacterial activity, could thus offer a possible alternative to antibiotics [15,16,17,18,19,20,21,22,23,24,25,26,27,28].

The antioxidant and antimicrobial ability of LY is structure-dependent, as it is provided in a free form via dietary supplementation. Therefore, avoiding LY degradation and maintaining structural stability during the gastrointestinal passage is key. Various approaches have been proposed to bypass degradation problems [29].

Among others, the binding of the bioactive molecule to a suitable carrier via an adsorption process has been proposed and would seem to be a viable, simple, and cost-effective solution [30,31,32].

Porous materials are some of the possible carriers that have been widely studied, because they can be customised to the specific application [33,34]. Clay minerals and zeolites are part of this materials group, and are already used in feed additives for other purposes [35,36].

When a clay mineral is used as a carrier, the intercalation process is of primary importance in modulating and controlling the availability of the organic molecule [37,38,39,40]. Among the different clays available in nature [39,40], montmorillonites have been reported as the most fit for purpose [32,35,36,41,42,43].

Although adsorption is a simple process, the interaction of a bioactive molecule with the porous matrix is complex. In fact, the molecular mobility in porous media is strongly dependent on the nature of the carrier, namely its porosity, and on the nature of the medium where the biomolecule is spread [44,45,46,47,48]. In fact, many molecule–carrier interactions are possible, such as size-dependent steric interactions, Van der Waals interactions, electrostatic interactions, and/or interactions mediated by solvents when present [33].

The adsorption process as well as the final properties of the synthesised materials are thus strongly influenced by the reaction conditions, such as concentrations, pH, ionic strength, temperature, reaction environment, and time. Accordingly, many efforts have been made to understand and model the interactions between porous materials and biomolecules, e.g., large proteins such as LY [49,50,51,52].

Although of high quality, studies in the literature are generally performed either on a model, or on simplified systems where the adsorption reaction is performed on simplified carriers [52]. However, we believe that more studies on commercial feed components that are already available on the market and permitted by regulations are required if the aim is to promote a greener and more sustainable feed on the market.

In fact, introducing new components for feed improvement requires a lot of effort and time, as approval has to be gained for selling and marketing the new feed, which needs to comply with regulations [53]. The present paper thus aims to demonstrate the feasibility of a simple, environmentally friendly, and cost-effective synthesis of organoclays, where the carrier is a complex commercial clay- and zeolite-based mixture, i.e., Adsorbo^®^, and lysozyme is the bioactive molecule.

In our previous work [32], the LY adsorption via a solid/liquid process in an aqueous environment was assessed on bentonite, sepiolite, and Phil75^®^ zeolite. It was demonstrated that the pore dimensions, but above all the zero-point charge (ZPC) were the parameters controlling the LY–carrier interaction. Depending on the carrier nature and the operating conditions, one of the above two parameters prevailed over the other. The final LY–carrier interaction was the result of the preparation procedure, enabling the LY to be homogeneously spread onto the carrier surface [32].

A pH-dependent LY release was also found, and the observed higher LY release at pH 7 indicated the possibility of overcoming the gastric barrier to reach the intestinal environment [32]. The total LY release (0.3–0.5% *w*/*w*) observed in bentonite- and zeolite-based systems, combined with their response to pH, made these materials more appropriate for LY supply and modulation [32].

Adsorbo^®^, the carrier studied here, is a mixture of the above clays and zeolite, with 15% *w*/*w* bentonite, 15% *w*/*w* sepiolite, and 70% *w*/*w* of Phil 75^®^ zeolites. It has already been approved as a feed additive, and is commonly used in zootechnical practices, mainly for mycotoxin adsorption [54]. However, there is no way of establishing in advance whether the properties of the mixture, i.e., Adsorbo^®^, are the mere sum of the components or whether there is some synergic or adverse effect, when they are co-present.

This paper assessed the Lysozyme-Adsorbo^®^ organoclay synthesis. The solid/liquid route, which we previously developed for single components [31,32] was applied, and the effects of the operating conditions for an effective lysozyme loading onto the carriers were studied. Synthesised materials were fully characterised to obtain information on the LY–carrier interaction mechanism and their effect on the final organoclay properties.

The novelty of this paper lies in the use of the very simple, cost-effective and environmentally friendly solid/liquid adsorption approach, natural components, and aqueous environment, as well as the use of a commercial carrier, already applied in the practice, thus closer to the infield application.

## 2. Materials and Methods

### 2.1. Materials

An application of 99.9% pure powder of commercial hen egg white Lysozyme, (LY) (CAS RN 12650-88-3, supplied by Sigma Aldrich, St. Louis, MO, USA) was used. This is characterised by a molecular weight (MW) of 14.4 kDa, and an isoelectric point (IP) of +11.35, and it remains positively charged below the pKa [55].

Adsorbo^®^ (AD) was selected as the carrier (supplied by Biomicron s.r.l Reggio Emilia, RE, Italy), and is a commercial mixture of clays (15% *w*/*w* bentonite, 15% *w*/*w* sepiolite, 70% *w*/*w* zeolite Phil75^®^), which is commonly used in zootechnical applications.

Other chemicals, such as pure HNO_3_, NaOH (all supplied by Sigma Aldrich, St. Louis, MO, USA, 98% pure), and distilled water were also applied.

### 2.2. Absorption Procedure

**Figure 1:**
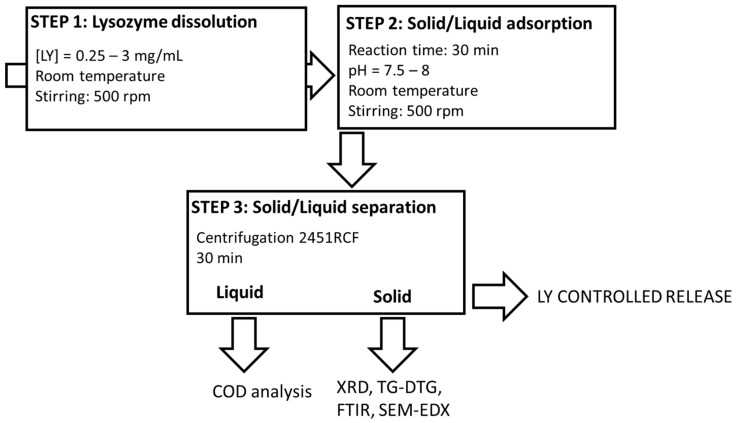
Lysozyme–Adsorbo^®^ adsorption process.

The final organoclays (LY-AD) were prepared according to the solid–liquid adsorption procedure (Figure 1), developed elsewhere for similar compositions [31,32,56].

Briefly, fixed amounts of LY were dissolved in 50 mL of demineralised water (pH = 3.8–4.3) and stirred at a rate of 500 rpm at room temperature until dissolution. Subsequently, 2 g of AD were added to the LY solution, and the suspensions were stirred at 500 rpm at room temperature for a fixed time. During the experiments, the pH was monitored (Mettler Toledo FE20/EL20 digital pH-meter; Mettler Toledo, Milan, MI, Italy), but not modified, because it naturally evolved and remained almost constant in the range 7.5–8. The effect of LY initial concentration was tested in the range 0.25–3 mg_LY_/mL, (i.e., 6.25–75 mg_LY_/g_AD_), while that of the reaction time was tested in the range 10–90 min.

Solid/liquid separation was then performed by centrifugation at 3000 rpm, (2451 relative centrifugal force, RCF) for 30 min (32 RotoFix centrifuge; Hettich Italia, Milano MI, Italy), and the solids and liquids were characterised.

All the synthesis was performed in mild conditions, i.e., an aqueous environment, and room temperature.

A mechanical mixture (LY-AD-MM) was also prepared by mixing, in an agate mortar, 2 g of the carrier with 75 mg of lysozyme, corresponding to a concentration solution of 1.5 mg/mL.

### 2.3. Characterisation

#### 2.3.1. Liquid Analysis

Before and after the solid/liquid adsorption experiments, solutions were analysed by chemical oxygen demand (COD) to determine the lysozyme content. Analyses were performed by HI80 Spectrophotometer (Hanna Instrument, Villafranca Padovana (PD), Italy), according to [57,58]. COD analyses were performed in triplicate, and the analytical error was ±2 mg_Ly_/g_AD_.

Captured LY was determined according to Equation (1):

LY_*capt*_ (mg_LY_/g_AD_) = [LY_*ini*_ (mg_LY_/g_AD_) − LY*_res_* (mg_LY_/g_AD_)] (1)

where LY_*capt*_ is the amount of captured lysozyme, LY_*ini*_ is the amount of lysozyme in the initial solution, and LY*_res_* the residual lysozyme in the solution after the reaction.

#### 2.3.2. Solids Characterisation

The morphology of the AD carrier material was assessed in terms of both the particle dimensions and porosity. Particle dimensions were determined by laser granulometry (CILAS 1180 granulometer; Orléans, France), according to [59,60]; meanwhile, the surface area (SA), pore volume (PV,) and pore dimensions (PD), were analysed with nitrogen adsorption at 77 K (Micromeritics Tristar 3000 Instrument; Micromeritics, Norcross, GA, USA) and Hg intrusion (Autopore V9600 Equipment, Micromeritics Instrument Corporation, Norcross, GA, USA). Before the analysis, the samples were degassed overnight at 60 °C (heating rate from 25 °C to 60 °C, 1 °C/min).

Phase composition of all the samples was determined by X-ray Powder Diffraction (XRPD), using a Panalytical Empyrean X-ray diffractometer, Bragg-Brentano geometry (Malvern Panalytical Ltd., Malvern, UK) operating at 40 kV/40 mA, and a large Nickel-beta filter, CuKα radiation, and a PIXcel3D detector. Patterns were collected in the 2θ range 2–70°, step size 0.0263° and counting time 356 s/step. Phase identification was performed using PANalytical B.V. software HIGHScore Plus v. 4.6a based on the ICSD database (FIZ Karlsruhe, Eggenstein-Leopoldshafen, Germany, ICSD web v. 2.1.0).

Thermal decomposition behaviour and LY–carrier interaction were analysed by thermogravimetric and differential thermogravimetric analyses (TG-DTG). Analysis was carried out in air by a DTA-TG SEIKO 6300 thermal analyser (Seiko Instruments Inc., Chiba, Japan) under the following experimental conditions: heating rate of 5 °C/min, T range = 25–800 °C.

Functional groups present in all the samples were assessed via skeletal spectra by the Fourier transform-infrared (FT-IR) spectroscopy on KBr–pellets (about 1% wt of sample powder) using a Thermonicolet Nexus (Thermo Fisher, Waltham, MA, USA) in the range 4000–400 cm^−1^ with 100 scans per analysis and 4 cm^−1^ resolution. The OMINC software (v. 7.2) for spectra analysis was also used. Surface functional groups were also determined by spectra in attenuated total reflectance (ATR) mode recorded with the same instrument equipped with an ATR accessory (diamond window).

Surface charge of the carrier was determined by zero point charge (ZPC) by means of a Zetasizer Nano ZS (Malvern Instruments Limited, Malvern, UK). Dynamic light scattering (DLS) at 90°, with a non-invasive backscatter (NIBS), was used for the measurements.

Sample morphology and elemental composition, as well as lysozyme dispersion in the organoclays, were analysed by scanning electron microscopy and energy dispersion X-ray spectroscopy (SEM-EDX) analyses by means of a Zeiss EVO 50 EP (Zeiss, Jena, Germany) combined with an Oxford INCA energy 2000 spectrometer (Oxford Instruments, Abingdon, UK). The SEM-EDX equipment was operated at an electron high tension (EHT) voltage of 15 and 20 kV, a probe current of 120 and 300 pA, and at high vacuum (about 10^−4^ Pa) conditions.

#### 2.3.3. Preliminary LY Release Tests on the LY-AD Samples

**Figure 2:**
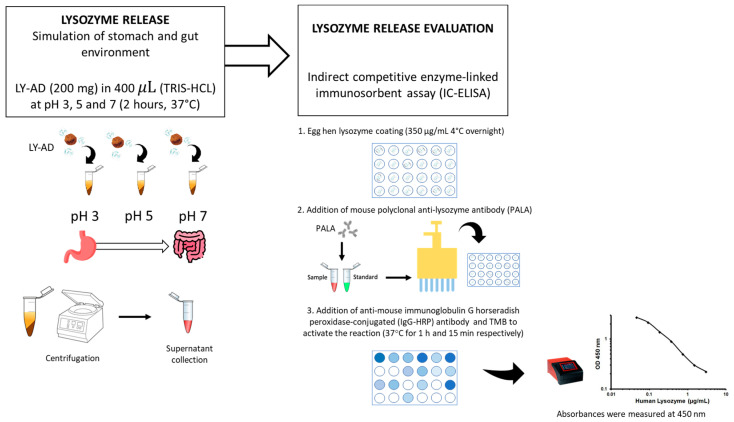
Methodology workflow for the preliminary lysozyme release assay.

To verify whether the synthesised organoclays (LY-AD) were able to release lysozyme in different environments, preliminary tests of lysozyme release were performed at different pH values, simulating stomach and gut environments, using the procedure shown in Figure 2.

Briefly, 200 mg of each LY–carrier powder was added to 400 μL of 10 mM tris(hydroxymethyl)aminomethane hydrochloric acid (TRIS-HCl; supplied by Sigma Aldrich, St. Louis, MO, USA) at pH 3, pH 5, and pH 7. The mixture was then shaken in a rotatory shaker at 180 revolutions per minute (rpm) for 2 h at 37 °C. The supernatant was then separated by centrifugation at 12,000 rpm (16,128 relative centrifugal force, RCF) for 10 min. The liquid extracts were stored at −20 °C until the next assay. The lysozyme release was quantified by indirect competitive enzyme-linked immunosorbent assay (IC-ELISA). Hen egg-white lysozyme (supplied by Sigma Aldrich, St. Louis, MO, USA) was coated (350 μg/mL in a solution of CaCO_3_ 50 mM) onto a 96 micro-well plate overnight at 4 °C, after which the plate was washed three times with 0.01 M phosphate-buffered saline at pH 7 (PBS; Sigma Aldrich, St. Louis, MO, USA), and then blocked with 200 μL of 1% (*w*/*v*) bovine serum albumin (BSA; supplied by Sigma Aldrich, St. Louis, MO, USA) for 2 h at 37 °C. 

The plate was then washed with 0.01 M PBS containing 0.05% (*v*/*v*) Tween 20^®^ (PBS-T; supplied by Sigma Aldrich, St. Louis, MO, USA). A total of 100 μL of mouse polyclonal anti-lysozyme antibody (supplied by Santa Cruz Biotechnology, San Juan, CA, USA) diluted 1:3000 was mixed with 100 μL of each sample. A total of 100 μL solution from each mixture was added to each coated well, and the plate was incubated at 37 °C for 1 h. The plate was washed with PBS-T and then a 1:10,000 diluted solution (100 μL) of anti-mouse immunoglobulin G horseradish peroxidase-conjugated (IgG-HRP) antibody (supplied by Sigma Aldrich, St. Louis, MO, USA) was added to each well and incubated at 37 °C for 1 h. The plate was then rewashed with PBS-T, and 50 μL of 3′,5,5-tetramethylbenzidine (TMB; supplied by Sigma Aldrich, St. Louis, MO, USA) was added to each well followed by incubation at 37 °C for 15 min. To stop the reaction, 150 μL of 0.4 N hydrochloric acid was added to each well, and then the absorbance was measured at 450 nm using a microplate reader (model 680; Bio-Rad, Hercules, CA, USA). Each sample was tested in triplicate. To obtain the standard curve, a recombinant egg lysozyme (supplied by Sigma Aldrich, St. Louis, MO, USA) was used. Six different concentrations ranging from 0 to 20 mg/mL in TRIS 10 mM were measured. The lysozyme release was expressed as a percentage of the total content considering 100% release as the maximum quantity of lysozyme present in the analysed organoclay.

#### 2.3.4. Preliminary Antioxidant Capacity Determination

A Trolox equivalent antioxidant capacity (TEAC) assay was adopted to evaluate the antioxidant effect of hydrolysed lysozyme, and tested as follows.

**Figure 3:**
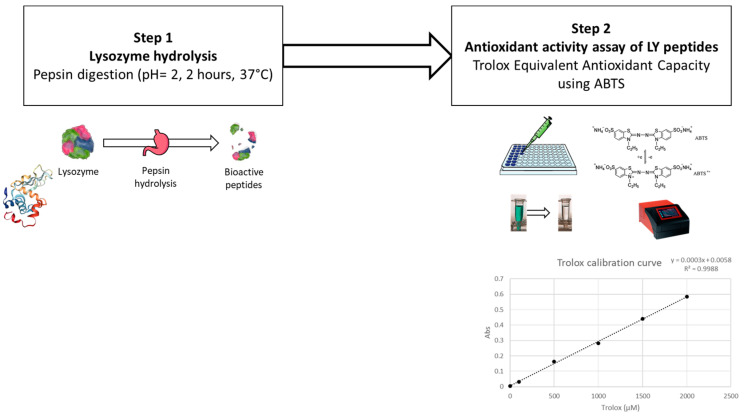
Steps in the preliminary antioxidant activity assay of lysozyme.

Firstly, lysozyme was hydrolysed by a pepsin digestion according to Memarpoor-Yazdi et al., (2012) [15], with slight modifications (Figure 3). Briefly, 0.5 mg of pepsin (P7000, ≥250 units/mg solid; supplied by Sigma-Aldrich, St. Louis, MO, USA) in Tris(hydroxymethyl)aminomethane buffer (50 mM, pH 2.0) (supplied by Sigma-Aldrich, St. Louis, MO, USA) were added to 10 mg of lysozyme (using an enzyme–substrate ratio of 1:20 *w*/*w*) and incubated at 37 °C for 2 h. The hydrolysed lysozyme was further centrifuged at 7000× *g* for 10 min, and the supernatant was filtered through a 20 µm syringe filter for further evaluation.

For the TEAC analysis, a 2,2′-azino-bis (3-ethylbenzothiazoline-6-sulfonic acid) (ABTS; supplied by Sigma-Aldrich, St. Louis, MO, USA) radical cation decolourisation test was performed as described by You et al. (2010) [61]. ABTS^•+^ was generated by diluting potassium persulfate in ABTS (7 mM) to obtain a final concentration of 2.45 mM. The reaction was left to stand in the dark for 16 h at room temperature. ABTS^•+^ was diluted in MilliQ water to obtain the working solution with an optical density (OD) of 0.70 ± 0.02 at 734 nm at room temperature. A concentration series (from 30 to 2 µM) of Trolox (6-hydroxy-2,5,7,8-tetramethychroman-2-carboxylic acid; supplied by Sigma-Aldrich, St. Louis, MO, USA) was used to build the calibration curve. Samples and standards were tested by mixing 100 µL with 100 µL of diluted ABTS^•+^ working solution in a 96-microwell plate. ODs were recorded using a microplate spectrometer at 734 nm after 8 min of reaction (BioTek Epoch Microplate Spectrophotometer; Agilent Technologies, Santa Clara, CA, USA). Percentage inhibition (PI) was calculated according to Equation (2):

PI = [(AbsABTS^•+^ − Abs sample)/AbsABTS^•+^] × 100 (2)

where AbsABTS^•+^ is the initial OD of diluted ABTS^•+^ and Abs indicates the final absorbance after 8 min of reaction. The Trolox calibration curve was used to express data as µmol of Trolox equivalents on a gram of hydrolysed enzyme (µmol TroloxEq/g).

Hereafter, abbreviations are used to identify both the organoclays and pristine materials. All the organoclays are identified by a label containing the actual LY loading per gram of carrier; for example, LY(32)-AD corresponds to the organoclay loaded with 32 mg of LY per gram of AD.

For the sake of clarity, abbreviations are spelled out in their full form in Table S1. The same table also shows a list of the characterisation techniques and their abbreviations, as well as those of the most complex reactants.

In addition, according to [62], “s” stands for “second”, “min” stands for “minute”, and “h” stands for “hour”. 

## 3. Results

### 3.1. Operating Parameters of the Organoclay Synthesis: Reaction Time, Initial Lysozyme (LY) Concentrations, and pH

The effects of the operating parameters on the LY-AD organoclay synthesis were fully explored in order to optimise the solid/liquid adsorption procedure, and to obtain a final LY loading of 37.5 mg_LY_/g_AD_ on the AD carrier, reported in the literature as being appropriate for nutritional zootechnical applications (target of supply 100 mg of LY per kg of diet) [63].

**Figure 4:**
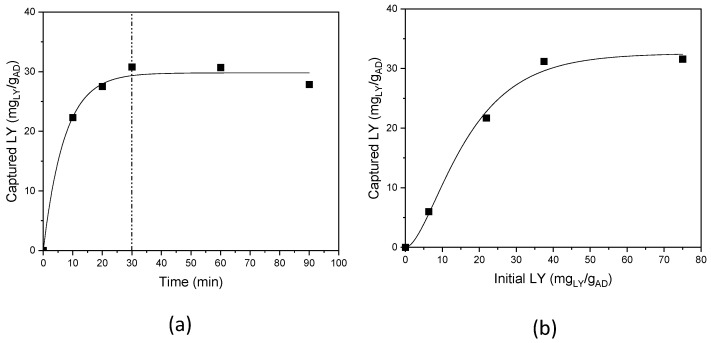
LY capture: (**a**) as a function of reaction time, and (**b**) as a function of the initial LY content.

The influence of the reaction time, was studied in the range 10–90 min, fixing the LY initial concentration at 1.5 mg/mL (i.e., 37.5 mg_LY_/g_AD_), pH 7.5–8; and room temperature (Figure 4a) [32].

LY capture is time-dependent; a progressive increase in the uptake was observed up to 30 min of reaction time, after which a plateau was reached, and a maximum and constant LY loading of 32 mg_LY_/g_AD_, accounting for 85% reaction yield, was achieved. Thus, the experiments were subsequently performed with 30 min as the reaction time.

The dependence on the initial LY concentration was investigated, in the range 0.25–3.0 mg/mL (corresponding to 6.25–75 mg_LY_/g_AD_) (Figure 4b). Again, a plateau trend was observed for the initial LY concentrations, and a maximum LY loading of 32 mg_LY_/g_AD_, was achieved for an initial LY content of 37.5 mg_LY_/g_AD_, and no loading increase was obtained, also doubling the initial LY content; an initial LY content of 37.5 mg_LY_/g_AD_ was thus used.

The pH effect [32,64,65] was studied in the range 2–11.3, (LY initial content 37.5 mg_LY_/g_AD_ and 30 min of reaction time), also considering pH = 4.3, i.e., no correction of the pH of the LY solutions [32]. Constant LY loading, in the range 31–32 mg_LY_/g_AD_, was obtained throughout the experimental pH range (Figure S1 in Supplementary Materials). All the experiments were thus performed at a pH in the range 7.5–8 only by monitoring and not correcting the pH.

In summary, the correct operating conditions to obtain the maximum LY loading (32 mg_LY_/g_AD_), which is very close to the target (37.5 mg_LY_/g_AD_), are:

- Initial LY content = 37.5 mg_LY_/g_AD_.
- pH = 7.5–8 (no pH correction).
- Reaction time = 30 min.
- Room temperature.
- Aqueous environment.

### 3.2. Characterisation of Pristine Lysozyme (LY) and Adsorbo^®^ (AD) Carrier

#### 3.2.1. Lysozyme (LY) Characterisation

LY is an amorphous powder. Its molecule is characterised by amide bonds whose principal IR bands (not reported) fall at 1655 cm^−1^, 1548 cm^−1^ and around 1300 cm^−1^ mainly attributed to the vibrational mode C=O stretching vibration (Amide I), the combination of N-H in plane-bending vibration and CN stretching mode (Amide II) and the combination of NH bending and CN stretching vibration (Amide III), respectively [32]. Thermal decomposition in air (by TG-DTG analysis, not reported) occurs in three different temperature regions, specifically 23–100 °C, corresponding to the physiosorbed water loss; 200–600 °C, with a maximum at 300 °C, where the lysozyme decomposition occurs; and 600–800 °C, due to the decomposition of the last organic residues [32].

#### 3.2.2. Adsorbo^®^ (AD) Characterisation

The commercial mineral mixture used as a carrier is a yellowish powder, characterised by particles with approximate dimensions of 2, 10, and 50 µm. By N_2_ adsorption, a surface area of 78 m^2^/g, a pore volume of 0.05 cm^3^/g, and mean pore diameters of 3 nm were determined. Adsorbo^®^ is characterised by a very negatively charged surface, with its ZPC equal to −141 (mV).

The carrier XRPD analysis (Figure S2 in Supplementary Materials) clearly identified peaks of phillipsite [ICSD 98-005-1639], chabazite [ICSD 98-015-6247], and zeolite [ICSD 98-028-1755]. The wide band in the 4–8° 2θ range, indicative of poor crystallinity, meant that the contribution of different clay minerals, such as montmorillonite, sepiolite, chlorite, and vermiculite could not be identified. Feldspar and dolomite were also present.

The very complex FT-IR spectrum of AD (Figure S3 in Supplementary Materials) is a result of the superimposition of bands due to the different mineral phases detected by the XRPD analysis. The strong band centred at 1030 cm^−1^ is characteristic of all silicate-based materials and is assigned to Si–O–Si vibrational modes, together with the broad shoulder at about 450 cm^−1^ [66]; weaker and broad bands in the range of 570–635 cm^−1^ and at about 510 cm^−1^ are consistent with the presence of zeolite [67]. The absorption near 500 cm^−1^ and the shoulder at 915 cm^−1^ are indicative of the vibrational modes of montmorillonite [68]. The bands at 1453, 884, and 730 cm^−1^ are coherent with the presence of dolomite in the sample as also provided by the XRPD analysis [69]. 

In the high frequency spectral region, the two weak bands at 3695 and 3620 cm^−1^ are due to stretching modes of structural OH groups, as also confirmed by the spectrum in ATR mode recorded to minimise the broad band due to water adsorbed on KBr matrix (Figure S3, inset). Adsorbed water, as well as the interlayer water of clay minerals, gives rise to the broad absorption centred at 3350 cm^−1^ and tailing to lower frequencies, corresponding to the deformation mode centred at 1645 cm^−1^.

TG and DTG analyses in the range 25–900 °C (Figures S4 and S5 in Supplementary Materials) highlighted three main decomposition temperature phenomena accounting for a total weight loss of 15% (*w*/*w*):

(I) Decomposition in the T range 25–100 °C, 4% weight loss, related to the water adsorbed on the surface of the clay minerals [70];
(II) Decomposition in the T range 100–200 °C, two complex and overlapped phenomena, 6% total weight loss, which is due to the zeolitic and crystalline water release in zeolite, as well as the interlayer water release in expandable clay minerals [70];
(III) Decomposition in the T range 400–700 °C, two steps, at above 450 °C and 650 °C, 5% total weight loss, accounting for sepiolite dehydration [70].

### 3.3. Characterisation of the Synthesised Organoclays at Increasing Lysozyme Loadings

Samples are identified by an acronym (listed in Table S1 in Supplementary Materials), where the composition is summarised; for example, LY(6)-AD, corresponds to the organoclay with 6 mg of lysozyme (LY) loading per gram of Adsorbo^®^ (AD).

#### 3.3.1. Study of Organoclay Phase Composition

The XRPD patterns (Figure S6 in Supplementary Materials) show a correlation between the lysozyme content and the intensity of the Bragg peaks of the zeolite at 2θ 26.5–28.5°. The intensity change is not accompanied by a variation in the unit cell edges, since the peak shift is negligible.

**Figure 5:**
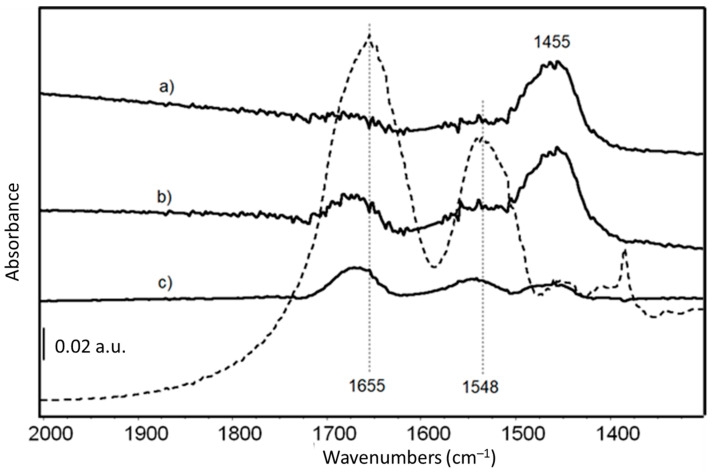
FT-IR skeletal spectra of (**a**) LY(6)-AD; (**b**) LY(22)-AD; (**c**) LY(32)-AD, upon subtraction of spectrum of pristine AD. Dashed line: FT IR skeletal spectrum of pure LY in KBr.

FTIR skeletal spectra of LY-AD organoclays at different LY loadings, upon subtraction of pristine AD spectrum, are shown in Figure 5, in comparison with the spectrum of pure LY. Due to the very weak signals of the organic moiety in the spectrum of the hybrid materials, the pure AD spectrum was subtracted from the spectra of the hybrid materials to enhance the bands characterising the LY structure.

The main bands are those previously assigned to Amide I and Amide II modes of the protein [71], slightly shifted to higher frequencies due to the interaction with the AD carrier.

Amide I and II bands are barely detectable in the samples at the lower LY content (Figure 5 spectra a, b), while their intensity increases with increasing LY loading (Figure 5 spectrum c). The band around 1455 cm^−1^, assigned to carbonate species, is reduced in the spectrum of LY(32)-AD (Figure 5 spectrum c), with respect to the samples with lower LY loadings (Figure 5). The carbonate bands are associated with the presence of dolomite, always detected by XRPD, and/or related to adsorbed carbonates due to the surface basicity of the clay.

At the high frequency region (Figure S7 in Supplementary Materials), two weak bands at 3620 cm^−1^ and 3695 cm^−1^, typical of OH groups in the AD structure, are still evident. However, they are superimposed on the broad band of adsorbed and interlayer water in clay minerals, and the bands of the NH groups of the protein, probably involved in H-bonds.

**Figure 6:**
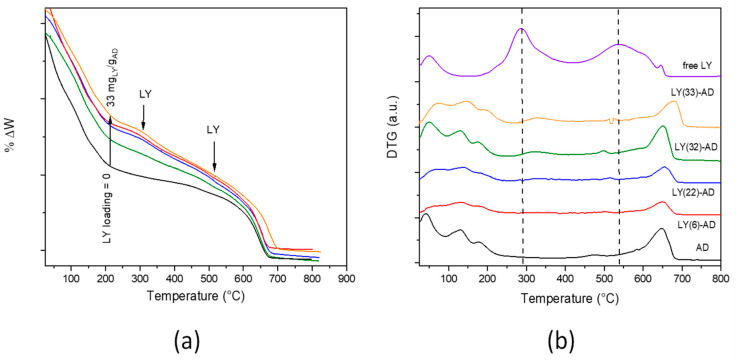
TGA (**a**) and DTG (**b**) curves of LY-AD samples at increasing LY loading (pristine AD and LY are reported for comparison, dashed lines: LY decomposition maxima). Curves were intentionally shifted to highlight differences; plotting in the original version in Figure S8 (Colours in (a) as in (b)).

In the TG curve (Figure 6a), the typical modulations of LY decomposition were barely distinguishable from those of the carrier. The weight loss of each decomposition step was calculated (Table S2 in Supplementary Materials). However, due to the superimposition of the different phenomena occurring in the pristine carriers, it was not possible to definitely attribute the total weight loss of approximately 7% (T range = 200–645 °C) to either LY or AD decomposition.

In the DTG analysis (Figure 6b) of LY-AD samples, the typical strong decomposition of pure lysozyme, maxima at 300 and 550 °C, was only barely evident. The broadening and the apparent shift of the maxima towards higher temperatures suggest an LY-AD interaction. In addition, in line with FTIR results, no physiosorbed water or coordinated water release in zeolites were clearly shown at 100–200 °C.

In summary, considering LY loading and phase composition, LY(32)-AD is the most interesting organoclay for the final application. It was therefore further characterised to obtain information on LY allocation, dispersion homogeneity, the nature and strength of the LY–carrier interaction, as these parameters seem to be fundamental in biomolecule protection and release. A comparison with a mechanical mixture, LY-AD-MM (Table S1 in Supplementary Materials) of the same composition as the organoclay was therefore carried out. In fact, LY-AD-MM was prepared by grinding the components in a mortar. The components, mixed simply in their pristine form, were thus expected to be characterised by no bonds other than weak interactions [32]. The results are reported below.

#### 3.3.2. Study of Lysozyme–Carrier Interaction and Lysozyme Allocation in LY(32)-AD

Lysozyme-Adsorbo^®^ interactions were studied by XRPD, FT-IR and DTG.

**Figure 7:**
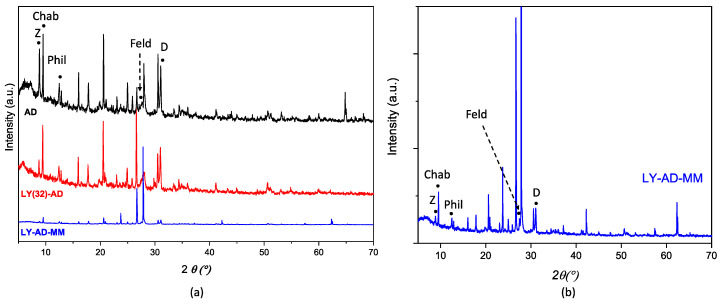
Comparison of XRPD patterns of (**a**) LY(32)-AD and LY-AD-MM, AD reported for comparison, and (**b**) enlarged pattern of LY-AD-MM. The position of the most intense (I_100_) reflections of the single phases is reported (Z: zeolite; Chab: chabazite; Phil: phillipsite; Feld: feldspar; D: dolomite).

XRPD patterns of AD, LY(32)-AD, and LY-AD-MM samples (Figure 7a,b) were quite similar. No shift in the reflection position was observed with respect to pristine AD, and only changes in the reflection intensities or broadening were detected. The variation in intensity in the zeolite pattern, (26.5–28.5° 2θ), seems to correlate with the presence of lysozyme, but without changes in the unit cell edges. In addition, the difference between the mixed samples and pristine AD could also be explained by the carrier inhomogeneity.

**Figure 8:**
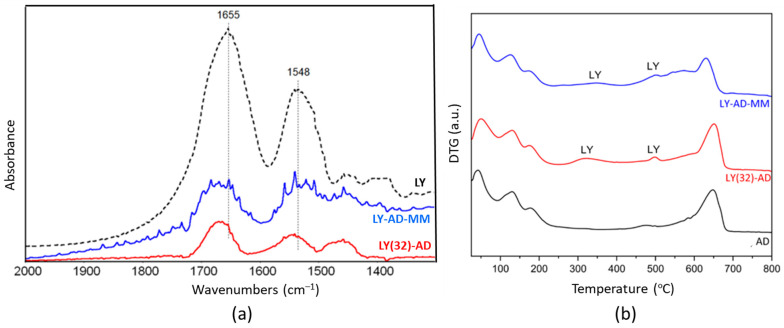
Comparison of the synthetised LY(32)-AD organoclay and the mechanical mixture LY-AD-MM: (**a**) FT-IR spectra upon AD subtraction, and (**b**) DTG. In both plots: LY-AD-MM in blue, LY(32)-AD in red. Dashed line in plot (**a**) pure LY spectrum.

The FT-IR spectrum (Figure 8a) of the mechanical mixture (blue line) highlighted the presence of the main amide bands, which overlapped significantly and were slightly broader than the spectrum of the corresponding LY(32)-AD organoclay.

This effect could indicate the presence of LY fractions that interact differently with the matrix, or also “free” LY. Residual carbonate species (band at 1450 cm^−1^ tailing to lower frequencies) were also still detected in the synthesised organoclay.

LY thermal decomposition (Figure 8b) in LY(32)-AD and LY-AD-MM occurred with similar trends, but at different temperatures than free LY. At the higher temperatures, the LY decomposition was detected at about 540 °C for both samples. The anticipated decomposition temperature, with respect to free LY (600–800 °C), suggests also a possible weak LY–carrier interaction for the mechanical mixture. 

**Figure 9:**
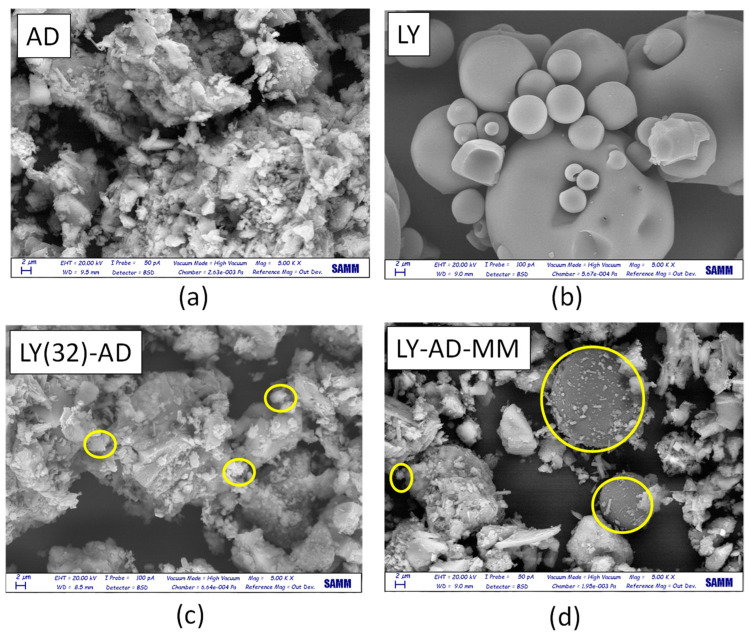
SEM images (magnification = 2 µm) of (**a**) pristine AD, (**b**) pristine LY, (**c**) LY(32)-AD the synthesised organoclay, and (**d**) LY-AD-MM the mechanical mixture. Yellow circles: LY aggregates.

Lysozyme allocation and spreading on the carrier surface were analysed by comparing SEM analyses of the pristine AD, free LY (Figure 9a,b), LY(32)-AD organoclay (Figure 9c), and the mechanical mixture LY-AD-MM (Figure 9d).

Pristine AD (Figure 9a), showed a complex morphology, highlighting the large and small aggregates, already detected by laser granulometry (2, 10, 50 µm). In contrast, free LY consisted of almost large or very large spherical aggregates, of different dimensions (Figure 9b).

In the SEM picture of the synthesised organoclay LY(32)-AD (Figure 9c), the characteristic features of the pristine carrier are predominant, only very small spherical LY aggregates (approximatively less than 2 µm) are barely identifiable (yellow circle in Figure 9c), thus suggesting a high LY dispersion.

The SEM picture of LY-AD-MM (Figure 9d) is very different, where the AD features and large spherical LY particles are clearly observable. LY aggregates, larger than 2 µm, are very similar to those of free LY (yellow circle in Figure 9d). However, in some portions of LY-AD-MM, smaller spherical LY aggregates can be observed, suggesting a very limited, but possible, LY dispersion. In line with FT-IR indications, different LY aggregate dimensions are consistent with different degrees of LY dispersion in the samples.

**Figure 10:**
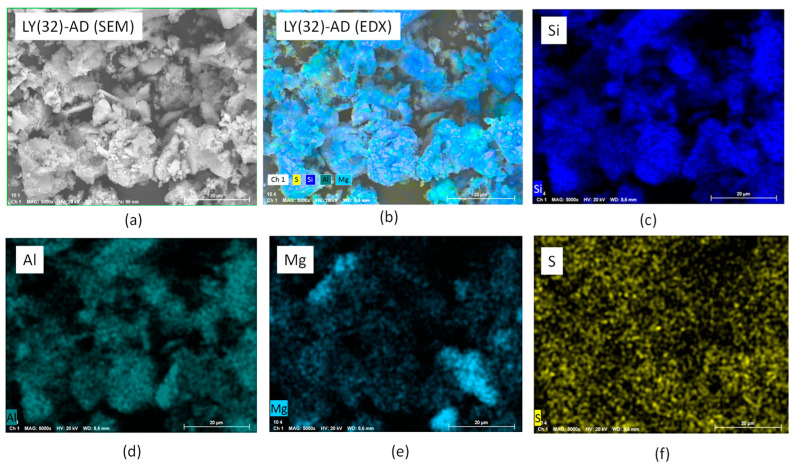
SEM-EDX analysis of LY (32)-AD organoclay (**a**) SEM of the analysed portion, (**b**) EXD of the analysed portion, (**c**) EXD of Si, (**d**) EDX of Al, (**e**) EDX of Mg, and (**f**) EDX of S.

To confirm this picture, EXD analyses were also performed. The results of LY(32)-AD are reported in Figure 10a–f, where the main carrier elements, Si, Mg, Al, were considered representative of the clay atomic components, as well as sulphur atoms as the LY tracer. Sulphur atoms were, in fact, present in the aminoacidic residues of the protein chain. The presence of Si, Al, and Mg (Figure 10c–e) was consistent with the carrier composition, while the presence of sulphur atoms, homogeneously dispersed in (Figure 10f), was a clear indication of the spread of LY molecules, thus interacting with AD, in line with both DTG and SEM analyses.

**Figure 11:**
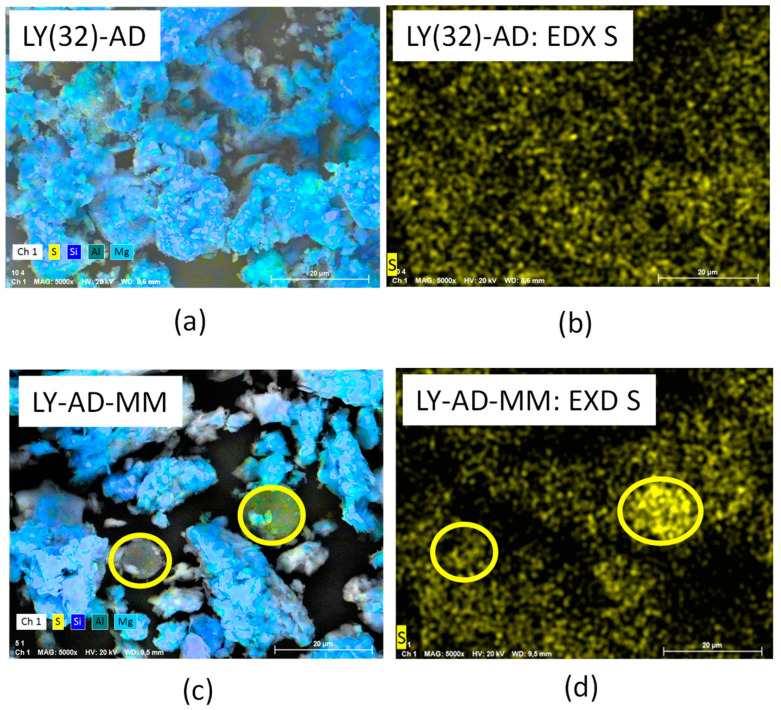
(**a**) SEM analysis of the synthesised organoclay, LY(32)-AD, (**b**) EDX analysis of the synthesised organoclay, LY(32)-AD, (**c**) SEM analysis of the mechanical mixture, LY-AD-MM, (**d**) EDX analysis of the mechanical mixture, LY-AD-MM. Yellow circles: lysozyme aggregates.

The higher LY dispersion in the synthetised sample was further confirmed by comparing the EDX of sulphur in LY(32)-AD organoclay and the EDX of sulphur in the mechanical mixture LY-AD-MM (Figure 11a–d).

In fact, in LY-AD-MM (Figure 11d) LY is only partially dispersed on the carrier surface, since it is mainly confined to the typical large spherical aggregates. Such large aggregates are no longer observable in LY(32)-AD organoclay (Figure 11b), where only dispersed LY molecules are present.

### 3.4. Preliminary Lysozyme Release Assay and Antioxidant Activity of the Hydrolysed Lysozyme

Releases at different pHs are summarised in Table 1, where the behaviour of the synthesised organoclay LY(32)-AD, and the corresponding mechanical mixture, LY-AD-MM, are compared.

In both samples, the release is pH-dependent. In the case of the synthesised organoclay, LY(32)-AD, the LY release progressively increases with increasing pH from 3 to 7. Conversely, in the mechanical mixture, LY-AD-MM, the highest release is observed at pH 3 while constant values are measured at pH 5 and 7. 

LY(32)-AD was also tested for the antioxidant capacity TEAC (Trolox equivalent antioxidant capacity) assay, and was also used to evaluate the antioxidant capacity of lysozyme after hydrolysis. Pepsin-digested polypeptides showed an antioxidant capacity of 1.47 ± 0.18 µmol TroloxEq/g with a percentage inhibition (PI) of 33.20 ± 3.94%.

## 4. Discussion

Our study of the operating parameters and our synthetic approach focused on LY binding to the AD carrier in order to optimise it for zootechnic applications. The aim was to protect biomolecules during the gastric passage, while ensuring the release of lysozyme into the intestinal tract.

Our synthetic approach—solid/liquid adsorption—is known to be effective in preparing organoclays [31,32,56], with the right features as those required for animal nutrition, such as well-dispersed LY loadings and reversible interactions with the carrier. The final result depends on the synthesis parameters [30,31,32]; i.e., the biomolecule concentration, pH, and reaction time, as well as the carrier characteristics, morphology, and the zero point charge.

Given the synthesis parameters discussed here, the maximum LY loading of 32 mg_LY_/g_AD_, is very close to the required target of 37.5 mg_LY_/g_AD_, and is reached by reacting the carrier with an aqueous solution containing 37.5 mg_LY_/g_AD_ at pH in range 7.5–8 for 30 minutes at room temperature.

Threshold loading is highlighted by the plateau behaviour (see Figure 4b), and likely indicates a site saturation mechanism as confirmed by the literature, where a site saturation was reported for all the AD components [32].

One of the determining factors for the applicability of these organoclays is the relative protein–surface orientation, because the wrong confinement and interaction negatively impact on the protein structure, and the related biological functions. The size and geometry of the hosting cavity and/or the nature of the binding surface [28,34,72] strongly influence the bioactivity of the adsorbate; therefore, knowledge of the nature and strength of the biomolecule–carrier interaction is of paramount importance.

Three key factors can explain the LY(32)–AD interaction mechanisms, based on the AD phase composition:

(1) LY physisorption in the pores of both zeolites and clay minerals [39,73];
(2) LY allocation in the interlayer of the expandable component (bentonite in this case), via an exchange reaction [31,74];
(3) LY interaction with the carrier surface, via charge interaction, related to ZPC of both LY and AD, and involving surface functional groups, such as hydroxyls [32,75].

These three mechanisms may act separately, jointly, or even in competition.

**Hypothesis 1.**  *Lysozyme adsorption due to morphology.*

The surface area and in particular the size of the pores of the sorbent control this process. The interactions of proteins, including LY, with cylindrical pores have been investigated by Sang and Coppens [76], considering mesoporous silica as a support, a SBA-15-based material, characterised by an average pore dimension of 6 nm. Cylindrical pores seemed to have a stabilising and protecting effect on the proteins. In Sang and Coppens, LY adsorption and activity were related to the surface curvature and the surface chemistry of the carrier. A similar behaviour was found by Kun-Che Kao et al. [72], for LY adsorption on silica at different levels of mesoporosity (average pore dimensions in the range 2.5–5.6 nm). Kun-Che Kao directly related the LY adsorption to pore dimension: the larger the pores, the larger the LY loading of the sample.

Hence, considering that the average pore diameter of Adsorbo^®^ is 3 nm, and the average volume of the typical LY ellipsoidal shape is 169 nm^3^ (calculated, considering the ellipsoid dimensions of 4.5 × 3.0 × 3.0 nm [77]), a large LY molecule cannot be accommodated inside small AD pores.

Although the adsorption inside the pores is less probable, it cannot be totally discarded. Indeed, the change in intensity of the zeolite Bragg peaks at 2θ 26.5–28.5°, reported for XRPD of the LY(32)-AD organoclay, may be associated with a slight variation in the local site chemistry, i.e., a change in the population of the cages of the phillipsitic (zeolitic) component of AD.

The adsorption process of LY on the sorbent could also lead to a partial denaturation of the globular protein [78], which can then partially occupy the cages of the crystalline structure of phillipsite. However, considering that no lysozyme degradation and/or hydrolyzation residues were clearly detected by FT-IR and are not expected considering the mild reaction conditions applied here, it is possible that only a LY–zeolite adsorption interaction took place.

**Hypothesis 2.**  *Lysozyme adsorption via ion exchange.*

To evaluate this hypothesis, recall that AD is a mixture of a zeolite (PH, Phil75^®^), bentonite (BN), and sepiolite (SP), where the zeolite is the main component (70% *w*/*w*).

In the case of exchangeable clay minerals, the interlayer cations, Na, Ca, or K [79], are prone to an exchange mechanism that is dependent on the replacing molecule and the operating conditions [64,80]. Thus, bentonite, the exchangeable clay AD component, should be able to intercalate LY in the interlayer via an exchange mechanism, which implies the release of Ca ions, and the XRD *d*_001_ reflection shift. 

In our organoclay samples, no clear shift in the diagnostic *d*_001_ was observed, as expected in cases of intercalation. However, the presence of an exchanging mechanism, although very limited, is suggested by the *d*_001_ broadening detected in the XRPD patterns of the organoclays at increasing LY content. The attempt to quantify exchanged Ca^2+^ ions during the adsorption reaction was prevented by the limited amount of bentonite (15% *w*/*w*) in the AD mixture [80]. Similar results were found when LY interactions with the individual components of the AD mixture were studied [32]. This evidence was explained with a very limited intercalation; at most, 0.576 mg of LY was exchanged per gram of bentonite, as calculated on the basis of the Ca^2+^ ions released during the process [32].

The negligible displacement of the interlayer water in DTG analyses of LY-AD samples, unexpected in cases of intercalation [64,80], would seem to indicate a very limited, if any, LY intercalation in the synthesised organoclay.

However, Amity Andersen et al. [47] reported how LY was captured through only a very limited intercalation, due to the interaction of the LY ammonium groups with the hydrated interlayer cations. Based on a dynamic modelling simulation of bentonite—an exchangeable clay mineral—Amity Andersen reported that the (010) edge surface was more chemically reactive than the basal (001) plane. A similar explanation could account for our evidence; thus, possible, although very limited, intercalation processes via interlayer cation exchange cannot be fully discarded for the LY(32)-AD organoclay. Furthermore, the presence of Ca^2+^ in solution, as a result of a limited intercalation process, is suggested by the different zeolite cage occupation, discussed above. Such changes could be a consequence of the uptake of a small amount of Ca^2+^ ions released by the bentonite component present in the Adsorbo^®^ mixture, which results in the intensity changes of the XRPD in the zeolite.

**Hypothesis 3.**  *adsorption via LY–surface site interaction.*

Lewis and Bronsted acid sites, present at the surface of clay minerals, can serve as binding centres for the lysozyme NH_2_ groups [65]. LY surface adsorption may also be affected by protein charge, size, stability, and aminoacidic composition. For hard proteins, such as LY, adsorption appears to be also favoured by hydrophobic surfaces, such as those of clay [48]. Finally, surface adsorption can be also driven by surface charge (i.e., ZPC) of the sorbent and of the adsorbate.

Adsorption via surface charge interaction could be very favoured if not even predominant, even in the presence of large external surface areas; at low temperatures (25 °C), LY cannot penetrate nanochannels, but it can be grafted onto the external surfaces [72]. Furthermore, positively charged lysozyme molecules are easily adsorbed on the negatively charged surface of silica-based sorbents in the pH range of 4.0−10.0 [72].

Our system appears to behave similarly. Our experimental conditions—pH 7.5–8 and room temperature—are typical in order to maintain LY positively charged [81]. When the positively charged LY molecules are put in contact with the strongly negative surface of Adsorbo^®^ (ZPC = −141 mV), the LY adsorption via the interaction of charges of opposite signs is promoted. This effect was already observed in previous work where the LY adsorption was performed on each individual component of AD [32], and confirmed by simulation results in the literature that show that electrostatic forces are mainly responsible for the LY–carrier binding process [45,46].

Our surface charge interactions are in line with the FT-IR results on LY(32)-AD, where a red shift in the protein bands was found. According to Balme et al. [82], such a shift is consistent with the interaction of lysozyme with the external surface of montmorillonite, and it was explained by a conformational change mainly due to dehydration [82].

In our organoclays, the lysozyme interaction with the clay surface is supported by much experimental evidence:

(a) a homogeneous distribution of sulphur atoms found by means of EDX analysis;
(b) the formation of H-bonds between the LY molecule and the inorganic matrix, not necessarily implying changes in conformation, as suggested by FT-IR analysis;
(c) the presence of carbonate species detected by XRPD and FT-IR analyses. Indeed, the presence of weaker carbonate bands in the FT-IR spectrum of LY(32)-AD, i.e., the sample with the maximum LY loading, seems to indicate a site saturation condition, as already highlighted by the plateau reached on increasing the concentration of LY during synthesis. The high LY dispersion, together with the LY steric hindrance, leads to a “crowded” surface of the carrier, which hampers CO_2_ adsorption, thus carbonate formation, and further LY loading;
(d) the broadening of the DTG modulation together with a lowering of the LY decomposition temperature, as supported by DTG analysis. This behaviour explains the decomposition of a large number of small LY aggregates.

Accordingly, the presence of such well-dispersed LY molecules highlights the strong interaction between AD and LY that derives from the interaction of charges of opposite signs, which is exploited via our preparation procedure.

Despite the very different LY dispersion, observed in SEM-EDX analysis, a very close decomposition temperature was found for LY(32)-AD and LY-AD-MM. However, the large and the broad decomposition at about 450–600 °C, observed in the DTG curve of LY-AD-MM, but not in LY(32)-AD, is consistent with the presence of “free” LY in the mechanical mixture. This finding is in line with our FT-IR spectra of the mechanical mixture, where LY molecules were found differently bonded.

The decomposition behaviour of the LY-AD-MM thus appears to be different from both a mere mechanical mixture of the components and the synthesised organoclay, suggesting the presence of an intermediate surface situation. Also, the interaction of charges of opposite signs and a strong affinity between LY and AD support this perspective.

Considering the three hypotheses discussed above, in all the synthesised organoclays, LY adsorption occurs mainly via the interaction of opposite charges, possibly involving the surface hydroxyls of the clay minerals and H of the lysozyme. Possibly, the surface adsorption is accompanied by a limited intercalation in bentonite or accommodation in zeolite cages. However, these phenomena may involve hydrolysed LY residues, aminoamides or small polypeptides, which are all smaller than the LY protein chain.

The charge interactions discussed here allow for a high LY dispersion, but are also probably responsible for site saturation.

The charge interaction mechanism may justify the considerable LY loading in LY(32)-AD organoclay. In fact, LY is captured in a zone “free” of restriction, unlike what would happen inside the pores. This capture mechanism may also account for the plateau trend in the adsorption, easily explained by a site saturation process due to a progressive neutralisation of charges. To explain site saturation, a possible steric hindrance among neighbouring molecules could also be considered.

As is clear from the FT-IR analysis of the organoclay, and according to the literature [46,47,48], LY is a “hard” protein, where no structural modification occurs during interactions with the sorbent. Therefore, each adsorbed LY molecule, due to its dimension, could occupy large surface portions, thus partially masking surface charge although not necessarily interacting with each other.

However, to better depict the surface situation, an in-depth surface characterisation is needed through dedicated characterisation techniques.

### Preliminary Release Tests and Antioxidant Capacity of the Hydrolysed Lysozyme

The presence of LY molecules allocated in different sites, bonded to different surface groups, intercalated or trapped inside the zeolite cages, could result in LY molecules that react differently to pH [83]. Accordingly, LY release and antioxidant capacity were preliminarily tested. These tests were performed solely to demonstrate that these materials could theoretically be used. This means that to be exhaustive, the tests would require further in-depth work which goes beyond the scope of this work.

A comparison of the release behaviour of the LY(32)-AD organoclay with that of the mechanical mixture, LY-AD-MM, revealed different trends in LY release, yet there was a common pH-dependence. In the LY(32)-AD organoclay, the lower the pH, the lower the LY release, with a maximum release value of 0.5% (*w*/*w*) at pH 7; at this pH, an absolute value of 0.16 mg_LY_/g_AD_ is released in 1 h.

Conversely, the mechanical mixture led to a greater LY release of 1.16 mg_LY_/g_AD_ at pH 5.

In conclusion, binding the LY to the carrier via our proposed synthetic approach could promote both LY protection and release during the gastrointestinal transit. LY release at a different pH in LY(32)-AD suggests the possibility of a controlled release in the different gastrointestinal tracts of the animal.

Although the antimicrobial activity of lysozyme has been known for decades, the antioxidant activity exerted by lysozyme in the host and its derived peptides has only been investigated recently and there is not much evidence of this effect. We thus only carried out preliminary and not exhaustive, release and antioxidant property tests. Our aim was to demonstrate the feasibility of applying these materials and so we focused on developing a new target delivery strategy for bioactive compounds, using lysozyme as a protein model. 

The ABTS assay revealed that LY hydrolysates possess a radical quenching ability. The values of TEAC that we found are in line with those of Memarpoor-Yazdi et al. [15], who identified the F2 peptide (NTDGSTDYGILQINSR) of hydrolysed egg-white lysozyme with antioxidant and antimicrobial potential. They showed that water soluble peptides with low molecular weight can react by scavenging the water soluble radical of ABTS, thus revealing higher activities compared with the lipid soluble systems used for antioxidant testing such as 2,2-diphenyl-1-picrylhydrazyl (DPPH) assay [61].

In our study, apart from the antioxidant activity, peptides released during the digestion significantly decreased the levels of oxidative markers (malondialdehyde) and increased the antioxidant enzymatic levels of superoxide dismutase, glutathione peroxidase, and total antioxidant capacity in weaned piglets’ serum [84]. Our results are thus in line with those encouraging the targeted delivery of bioactive compounds for disease prevention and enhancing the health status of animals.

Our findings indicate that clay-based materials could be used to release and supply bioactive molecules in the animal intestinal environment in order to develop functional diets for animal nutrition.

The supplementation of protected LY is thus promising. In addition, this supplementation extends the possibility for targeted delivery and release into the gastrointestinal tracts of other bioactive molecules in order to design functional diets. Our study thus encourages the development of precision nutritional technologies for the sustainable development of the livestock sector in line with all international policies.

Finally, these clay-based feed additives provide new opportunities for the controlled release and protection of bioactive molecules, given that they have a positive impact on both food safety and environmental protection in compliance with SDG2, SDG3, SDG6, SDG12, and SDG13.

## 5. Conclusions

In this paper, we have demonstrated that Adsorbo^®,^ is a simple, cost effective, and environmental synthetic approach for binding bioactive molecules, lysozyme, to natural carriers, and that it synthesizes hybrid materials characterised by peculiar properties.

Our synthetic route enables LY–carrier interactions, which result in LY molecules anchored to the carrier surface. This means that the carrier can exert both LY protection, and controlled LY release during application.

LY initial concentration and pH are the main parameters governing the synthetic process, together with the physico–chemical properties of the carriers and the bioactive molecules (e.g., morphology and surface nature).

The carriers’ zero point charge and morphology influence the adsorption process, more than phase composition, since the LY–carrier interactions are essentially due to charges of opposite signs.

Maximum LY loading of 32 mg_LY_/g_AD_ (corresponding to a yield of 85%) is obtained with a pH in the range 7.5–8, an initial LY content of 37.5 mg_LY_/g_AD_, and a 30-minute reaction time. At the end of the process, the LY molecules are spread relatively homogeneously over the carrier’s surface, where the LY-AD are different from those where the components are mixed.

Finally, in the hybrid material, the LY molecules are anchored to the carrier surface or edges. Consequently, both biomolecule protection and LY controlled release are active in different tracts of the intestinal environment. Accordingly, the in vitro approach here applied suggests our new targeted delivery strategy is feasible for bioactive compounds.

However, the applicability of the proposed technology, as well as the antioxidant and antibacterial effects, require further studies with a larger number of experiments and release kinetics. We believe that our simple and affordable synthetic approach could stimulate the development of improved functional feed formulations for sustainable applications in precision livestock farming.

## Figures and Tables

**Table 1:** Release percentages (*w*/*w*) and absolute values, Trolox equivalent antioxidant capacity (TAC) and percentage inhibition (IP), for the synthesised organoclay LY(32)-AD and the mechanical mixture LY-AD-MM.

Sample	LY Release at Different pH						TEAC	IP
	pH 3.57		pH 5		pH 7			
	(%)	mg_LY_/g_AD_	(%)	mg_LY_/g_AD_	(%)	mg_LY_/g_AD_	TroloxEq/g (μmol)	(%)
LY (32)-AD	0.2	0.064	0.3	0.096	0.5	0.16	1.47 ± 0.18	33.20 ± 3.94
LY-AD-MM	3.2	1.16	2.5	0.9	2.5	0.9	-	-

## Data Availability

Data are available upon request from the authors.

## Supplementary Materials

The following supporting information can be downloaded at: https://www.mdpi.com/article/10.3390/nano13222965/s1, Table S1: Explanation of the correspondence between abbreviation and full names of: samples, characterisation techniques, and the most complex reagents; Table S2: weight loss of each step of the thermal decomposition of the hybrid materials with different LY; Figure S1: captured LY as function of pH (2–11.3); Figure S2: XRPD analysis of pristine carrier Adsorbo^®^; Figure S3: FT-IR skeletal spectrum of Adsorbo^®^ in KBr; Inset: FT-IR spectrum of OH stretching region, spectrum recorded in ATR mode; Figure S4: TG analysis of pristine carrier: Adsorbo^®^ (AD); Figure S5: DTG curve of pristine Adsorbo^®^ (AD); Figure S6: XRPD patterns of the hybrid material at different LY loadings (AD: Adsorbo^®^, LY(X)-AD: hybrid samples at increasing LY loadings); Figure S7: FT-IR spectra of: (a) pristine AD, and (b) LY (32); Figure S8: Unshifted curves of TG of samples at increasing amounts of LY.

## References

[1] 32019R0006. Regulation (EU) 2019/6 of the European Parliament and of the Council of 11 December 2018 on Veterinary Medicinal Products and Repealing Directive 2001/82/EC (Text with EEA Relevance). https://eur-lex.europa.eu/eli/reg/2019/6/oj.

[2] Rhouma M., Fairbrother J.M., Beaudry F., Letellier A. (2017). Post Weaning Diarrhea in Pigs: Risk Factors and Non-Colistin-Based Control Strategies. Acta Vet. Scand..

[3] Patel M., Kumar R., Kishor K., Mlsna T., Pittman C.U., Mohan D. (2019). Pharmaceuticals of Emerging Concern in Aquatic Systems: Chemistry, Occurrence, Effects, and Removal Methods. Chem. Rev..

[4] Czech A., Nowakowicz-Debek B., Łukaszewicz M., Florek M., Ossowski M., Wlazło Ł. (2022). Effect of Fermented Rapeseed Meal in the Mixture for Growing Pigs on the Gastrointestinal Tract, Antioxidant Status, and Immune Response. Sci. Rep..

[5] Arias A., Feijoo G., Moreira M.T. (2022). Exploring the Potential of Antioxidants from Fruits and Vegetables and Strategies for Their Recovery. Innov. Food Sci. Emerg. Technol..

[6] Mnisi C.M., Mlambo V., Gila A., Matabane A.N., Mthiyane D.M.N., Kumanda C., Manyeula F., Gajana C.S. (2022). Antioxidant and Antimicrobial Properties of Selected Phytogenics for Sustainable Poultry Production. Appl. Sci..

[7] Frazzini S., Scaglia E., Dell’Anno M., Reggi S., Panseri S., Giromini C., Lanzoni D., Sgoifo Rossi C.A., Rossi L. (2022). Antioxidant and Antimicrobial Activity of Algal and Cyanobacterial Extracts: An In Vitro Study. Antioxidants.

[8] Wang L., Zhang Y., Liu L., Huang F., Dong B. (2021). Effects of Three-Layer Encapsulated Tea Tree Oil on Growth Performance, Antioxidant Capacity, and Intestinal Microbiota of Weaned Pigs. Front. Vet. Sci..

[9] Grossi S., Dell’Anno M., Rossi L., Compiani R., Sgoifo Rossi C.A. (2021). Supplementation of Live Yeast, Mannan Oligosaccharide, and Organic Selenium during the Adaptation Phase of Newly Arrived Beef Cattle: Effects on Health Status, Immune Functionality, and Growth Performance. Antibiotics.

[10] Dell’Anno M., Scaglia E., Reggi S., Grossi S., Angelo Sgoifo Rossi C., Frazzini S., Caprarulo V., Rossi L. (2023). Evaluation of Tributyrin Supplementation in Milk Replacer on Diarrhoea Occurrence in Pre-Weaning Holstein Calves. Animal.

[11] Mahfuz S., Shang Q., Piao X. (2021). Phenolic Compounds as Natural Feed Additives in Poultry and Swine Diets: A Review. J. Anim. Sci. Biotechnol..

[12] Ma X., Zhang S., Pan L., Piao X.S. (2017). Effects of Lysozyme on the Growth Performance, Nutrient Digestibility, Intestinal Barrier and Microbiota of Weaned Pigs Fed Diets Containing Spray-Dried Whole Egg or Albumen Powder. Can. J. Anim. Sci..

[13] Arsène M.M.J., Davares A.K.L., Viktorovna P.I., Andreevna S.L., Sarra S., Khelifi I., Sergueïevna D.M. (2022). The Public Health Issue of Antibiotic Residues in Food and Feed: Causes, Consequences, and Potential Solutions. Vet. World.

[14] Dragoni I., Balzaretti C., Rossini S., Rossi L., Dell’Orto V., Baldi A. (2011). Detection of Hen Lysozyme on Proteic Profiles of Grana Padano Cheese through SELDI-TOF MS High-Throughput Technology during the Ripening Process. Food Anal. Methods.

[15] Memarpoor-Yazdi M., Asoodeh A., Chamani J. (2012). A Novel Antioxidant and Antimicrobial Peptide from Hen Egg White Lysozyme Hydrolysates. J. Funct. Foods.

[16] Abu Hafsa S.H., Mahmoud A.E.M., Fayed A.M.A., Abdel-Azeem A.-A.S. (2022). The Effect of Exogenous Lysozyme Supplementation on Growth Performance, Caecal Fermentation and Microbiota, and Blood Constituents in Growing Rabbits. Animals.

[17] Zhang T., An W., Sun J., Duan F., Shao Z., Zhang F., Jiang T., Deng X., Boyer C., Gao W. (2022). *N*-Terminal Lysozyme Conjugation to a Cationic Polymer Enhances Antimicrobial Activity and Overcomes Antimicrobial Resistance. Nano Lett..

[18] Liang Q., Yuan M., Xu L., Lio E., Zhang F., Mou H., Secundo F. (2022). Application of Enzymes as a Feed Additive in Aquaculture. Mar. Life Sci. Technol..

[19] Wu T., Jiang Q., Wu D., Hu Y., Chen S., Ding T., Ye X., Liu D., Chen J. (2019). What Is New in Lysozyme Research and Its Application in Food Industry? A Review. Food Chem..

[20] Ibrahim H., Aoki T., Pellegrini A. (2002). Strategies for New Antimicrobial Proteins and Peptides: Lysozyme and Aprotinin as Model Molecules. CPD.

[21] Masschalck B., Van Houdt R., Haver E., Michiels C. (2001). Inactivation of Gram-Negative Bacteria by Lysozyme, Denatured Lysozyme, and Lysozyme-Derived Peptides under High Hydrostatic Pressure. Appl. Environ. Microbiol..

[22] Oliver W.T., Wells J.E. (2015). Lysozyme as an Alternative to Growth Promoting Antibiotics in Swine Production. J. Anim. Sci. Biotechnol..

[23] Ferraboschi P., Ciceri S., Grisenti P. (2021). Applications of Lysozyme, an Innate Immune Defense Factor, as an Alternative Antibiotic. Antibiotics.

[24] Vanrolleghem W., Tanghe S., Verstringe S., Bruggeman G., Papadopoulos D., Trevisi P., Zentek J., Sarrazin S., Dewulf J. (2019). Potential Dietary Feed Additives with Antibacterial Effects and Their Impact on Performance of Weaned Piglets: A Meta-Analysis. Vet. J..

[25] Nyachoti C.M., Kiarie E., Bhandari S.K., Zhang G., Krause D.O. (2012). Weaned Pig Responses to Escherichia Coli K88 Oral Challenge When Receiving a Lysozyme Supplement1,2. J. Anim. Sci..

[26] May K.D., Wells J.E., Maxwell C.V., Oliver W.T. (2012). Granulated Lysozyme as an Alternative to Antibiotics Improves Growth Performance and Small Intestinal Morphology of 10-Day-Old Pigs1. J. Anim. Sci..

[27] Wells J.E., Berry E.D., Kalchayanand N., Rempel L.A., Kim M., Oliver W.T. (2015). Effect of Lysozyme or Antibiotics on Faecal Zoonotic Pathogens in Nursery Pigs. J. Appl. Microbiol..

[28] Zou L., Xiong X., Liu H., Zhou J., Liu Y., Yin Y. (2019). Effects of Dietary Lysozyme Levels on Growth Performance, Intestinal Morphology, Immunity Response and Microbiota Community of Growing Pigs: Dietary Lysozyme Levels in Growing Pigs. J. Sci. Food Agric..

[29] Homayun B., Lin X., Choi H.-J. (2019). Challenges and Recent Progress in Oral Drug Delivery Systems for Biopharmaceuticals. Pharmaceutics.

[30] Giromini C., Tretola M., Cristiani C., Finocchio E., Silacci P., Panseri S., Dell’Anno M., Baldi A., Rossi L. (2021). Evaluation of the Absorption of Methionine Carried by Mineral Clays and Zeolites in Porcine Ex Vivo Permeability Models. Appl. Sci..

[31] Cristiani C., Finocchio E., Rossi L., Giromini C., Dell’Anno M., Panseri S., Bellotto M. (2021). Natural Clays as Potential Amino Acids Carriers for Animal Nutrition Application. Appl. Sci..

[32] Guagliano M., Dell’Anno M., Dotelli G., Finocchio E., Lacalamita M., Mesto E., Reggi S., Rossi L., Schingaro E., Staltari E. (2023). Lysozyme–Mineral Clay Systems: Comparison of Interaction for Controlled Release in Feed Application. Minerals.

[33] Huber P. (2015). Soft Matter in Hard Confinement: Phase Transition Thermodynamics, Structure, Texture, Diffusion and Flow in Nanoporous Media. J. Phys. Condens. Matter.

[34] Caetano D.L.Z., Metzler R., Cherstvy A.G., De Carvalho S.J. (2021). Adsorption of Lysozyme into a Charged Confining Pore. Phys. Chem. Chem. Phys..

[35] Nadziakiewicza M., Kehoe S., Micek P. (2019). Physico-Chemical Properties of Clay Minerals and Their Use as a Health Promoting Feed Additive. Animals.

[36] Subramaniam M.D., Kim I.H. (2015). Clays as Dietary Supplements for Swine: A Review. J. Anim. Sci. Biotechnol..

[37] Poyatos-Racionero E., Pérez-Esteve É., Medaglia S., Aznar E., Barat J.M., Martínez-Máñez R., Marcos M.D., Bernardos A. (2022). Gated Organonanoclays for Large Biomolecules: Controlled Release Triggered by Surfactant Stimulus. Nanomaterials.

[38] Șerban M.V., Nazarie S.-R., Dinescu S., Radu I.-C., Zaharia C., Istrătoiu E.-A., Tănasă E., Herman H., Gharbia S., Baltă C. (2022). Silk ProteinsEnriched Nanocomposite Hydrogels Based on Modified MMT Clay and Poly(2-Hydroxyethyl Methacrylate-Co-2-Acrylamido-2-Methylpropane Sulfonic Acid) Display Favorable Properties for Soft Tissue Engineering. Nanomaterials.

[39] Massaro M., Colletti C., Lazzara G., Riela S. (2018). The Use of Some Clay Minerals as Natural Resources for Drug Carrier Applications. JFB.

[40] Rodrigues L.A.D.S., Figueiras A., Veiga F., De Freitas R.M., Nunes L.C.C., Da Silva Filho E.C., Da Silva Leite C.M. (2013). The Systems Containing Clays and Clay Minerals from Modified Drug Release: A Review. Colloids Surf. B Biointerfaces.

[41] Damato A., Vianello F., Novelli E., Balzan S., Gianesella M., Giaretta E., Gabai G. (2022). Comprehensive Review on the Interactions of Clay Minerals with Animal Physiology and Production. Front. Vet. Sci..

[42] Liu J.H., Cai W.K., Khatoon N., Yu W.H., Zhou C.H. (2021). On How Montmorillonite as an Ingredient in Animal Feed Functions. Appl. Clay Sci..

[43] Adiga S.P., Jin C., Curtiss L.A., Monteiro-Riviere N.A., Narayan R.J. (2009). Nanoporous Membranes for Medical and Biological Applications: Nanoporous Membranes for Medical and Biological Applications. WIREs Nanomed. Nanobiotechnol..

[44] Patamia V., Zagni C., Fiorenza R., Fuochi V., Dattilo S., Riccobene P.M., Furneri P.M., Floresta G., Rescifina A. (2023). Total Bio-Based Material for Drug Delivery and Iron Chelation to Fight Cancer through Antimicrobial Activity. Nanomaterials.

[45] Kolman K., Makowski M.M., Golriz A.A., Kappl M., Pigłowski J., Butt H.-J., Kiersnowski A. (2014). Adsorption, Aggregation, and Desorption of Proteins on Smectite Particles. Langmuir.

[46] Kubiak-Ossowska K., Mulheran P.A. (2010). What Governs Protein Adsorption and Immobilization at a Charged Solid Surface?. Langmuir.

[47] Andersen A., Reardon P.N., Chacon S.S., Qafoku N.P., Washton N.M., Kleber M. (2016). Protein–Mineral Interactions: Molecular Dynamics Simulations Capture Importance of Variations in Mineral Surface Composition and Structure. Langmuir.

[48] Lepoitevin M., Jaber M., Guégan R., Janot J.-M., Dejardin P., Henn F., Balme S. (2014). BSA and Lysozyme Adsorption on Homoionic Montmorillonite: Influence of the Interlayer Cation. Appl. Clay Sci..

[49] Anglin E., Cheng L., Freeman W., Sailor M. (2008). Porous Silicon in Drug Delivery Devices and Materials. Adv. Drug Deliv. Rev..

[50] Tinsley-Bown A.M., Canham L.T., Hollings M., Anderson M.H., Reeves C.L., Cox T.I., Nicklin S., Squirrell D.J., Perkins E., Hutchinson A. (2000). Tuning the Pore Size and Surface Chemistry of Porous Silicon for Immunoassays. Phys. Status. Solidi..

[51] Salonen J., Kaukonen A.M., Hirvonen J., Lehto V.-P. (2008). Mesoporous Silicon in Drug Delivery Applications. J. Pharm. Sci..

[52] Hartmann M., Kostrov X. (2013). Immobilization of Enzymes on Porous Silicas–Benefits and Challenges. Chem. Soc. Rev..

[53] Coppens P., Da Silva M.F., Pettman S. (2006). European Regulations on Nutraceuticals, Dietary Supplements and Functional Foods: A Framework Based on Safety. Toxicology.

[54] Technical Data Sheet. https://biomicronfeed.com/wp-content/uploads/2015/12/scheda-tecnica-ADSORBO-alimenti-per-animali.pdf.

[55] Proctor V.A., Cunningham F.E., Fung D.Y.C. (1988). The Chemistry of Lysozyme and Its Use as a Food Preservative and a Pharmaceutical. CRC Crit. Rev. Food Sci. Nutr..

[56] Zampori L., Stampino P.G., Cristiani C., Cazzola P., Dotelli G. (2010). Intercalation of Poly(Ethylene-Oxides) in Montmorillonite: Tailor-Made Nanocontainers for Drug Delivery Systems. Appl. Clay Sci..

[57] O’Dell J.W. (1996). The Determination of Chemical Oxygen Demand by Semi-Automated Colorimetry. Methods for the Determination of Metals in Environmental Samples.

[58] 5220 Chemical Oxygen Demand (COD) (2017). Standard Methods for the Examination of Water and Wastewater.

[59] Klank D., Goverde T., Blum C. (2009). Particle World.

[60] Polakowski C., Ryżak M., Sochan A., Beczek M., Mazur R., Bieganowski A. (2021). Particle Size Distribution of Various Soil Materials Measured by Laser Diffraction—The Problem of Reproducibility. Minerals.

[61] You S.-J., Udenigwe C.C., Aluko R.E., Wu J. (2010). Multifunctional Peptides from Egg White Lysozyme. Food Res. Int..

[62] Kate Miller-Wilson B.A. Staff Abbreviations & Acronyms, Seconds, Minutes, and Hours: Abbreviations & Conversions. https://www.yourdictionary.com/articles/sec-min-hr-abbreviations-conversions.

[63] Oliver W.T., Wells J.E., Maxwell C.V. (2014). Lysozyme as an Alternative to Antibiotics Improves Performance in Nursery Pigs during an Indirect Immune Challenge1,2. J. Anim. Sci..

[64] Iannicelli-Zubiani E.M., Cristiani C., Dotelli G., Gallo Stampino P., Pelosato R., Mesto E., Schingaro E., Lacalamita M. (2015). Use of Natural Clays as Sorbent Materials for Rare Earth Ions: Materials Characterization and Set up of the Operative Parameters. Waste Manag..

[65] Kalburcu T., Tabak A., Ozturk N., Tuzmen N., Akgol S., Caglar B., Denizli A. (2015). Adsorption of Lysozyme from Aqueous Solutions by a Novel Bentonite–Tyrptophane (Bent–Trp) Microcomposite Affinity Sorbent. J. Mol. Struct..

[66] Lazarević S., Janković-Častvan I., Jovanović D., Milonjić S., Janaćković D., Petrović R. (2007). Adsorption of Pb^2+^, Cd^2+^ and Sr^2+^ Ions onto Natural and Acid-Activated Sepiolites. Appl. Clay Sci..

[67] Mozgawa W., Król M., Barczyk K. (2011). FT-IR Studies of Zeolites from Different Structural Groups. CHEMIK.

[68] Madejová J. (2003). FTIR Techniques in Clay Mineral Studies. Vib. Spectrosc..

[69] Ji J., Ge Y., Balsam W., Damuth J.E., Chen J. (2009). Rapid Identification of Dolomite Using a Fourier Transform Infrared Spectrophotometer (FTIR): A Fast Method for Identifying Heinrich Events in IODP Site U1308. Mar. Geol..

[70] Földvári M. (2011). Handbook of Thermogravimetric System of Minerals and Its Use in Geological Practice.

[71] Barth A. (2007). Infrared Spectroscopy of Proteins. Biochim. Et Biophys. Acta (BBA)–Bioenerg..

[72] Kao K.-C., Lin T.-S., Mou C.-Y. (2014). Enhanced Activity and Stability of Lysozyme by Immobilization in the Matching Nanochannels of Mesoporous Silica Nanoparticles. J. Phys. Chem. C.

[73] Villa C.C., Valencia G.A., López Córdoba A., Ortega-Toro R., Ahmed S., Gutiérrez T.J. (2022). Zeolites for Food Applications: A Review. Food Biosci..

[74] Zampori L., Dotelli G., Gallo Stampino P., Cristiani C., Zorzi F., Finocchio E. (2012). Thermal Characterization of a Montmorillonite, Modified with Polyethylene-Glycols (PEG1500 and PEG4000), by in Situ HT-XRD and FT IR: Formation of a High-Temperature Phase. Appl. Clay Sci..

[75] An N., Zhou C.H., Zhuang X.Y., Tong D.S., Yu W.H. (2015). Immobilization of Enzymes on Clay Minerals for Biocatalysts and Biosensors. Appl. Clay Sci..

[76] Sang L.-C., Coppens M.-O. (2011). Effects of Surface Curvature and Surface Chemistry on the Structure and Activity of Proteins Adsorbed in Nanopores. Phys. Chem. Chem. Phys..

[77] Cegielska-Radziejewska R., Lesnierowski G., Kijowski J. (2008). Properties and Application of Egg White Lysozyme and Its Modified Preparations—A Review. Pol. J. Food Nutr. Sci..

[78] Eş I., Vieira J.D.G., Amaral A.C. (2015). Principles, Techniques, and Applications of Biocatalyst Immobilization for Industrial Application. Appl. Microbiol. Biotechnol..

[79] Fil B.A., Özmetin C., Korkmaz M. (2014). Characterization and Electrokinetic Properties of Montmorillonite. Bulg. Chem. Commun..

[80] Cristiani C., Iannicelli-Zubiani E.M., Dotelli G., Finocchio E., Gallo Stampino P., Licchelli M. (2019). Polyamine-Based Organo-Clays for Polluted Water Treatment: Effect of Polyamine Structure and Content. Polymers.

[81] Kuehner D.E., Engmann J., Fergg F., Wernick M., Blanch H.W., Prausnitz J.M. (1999). Lysozyme Net Charge and Ion Binding in Concentrated Aqueous Electrolyte Solutions. J. Phys. Chem. B.

[82] Balme S., Guégan R., Janot J.-M., Jaber M., Lepoitevin M., Dejardin P., Bourrat X., Motelica-Heino M. (2013). Structure, Orientation and Stability of Lysozyme Confined in Layered Materials. Soft Matter.

[83] Chang Y.-K., Chu L., Tsai J.-C., Chiu S.-J. (2006). Kinetic Study of Immobilized Lysozyme on the Extrudate-Shaped NaY Zeolite. Process Biochem..

[84] Xu Q.-L., Liu C., Mo X.-J., Chen M., Zhao X.-L., Liu M.-Z., Wang S.-B., Zhou B., Zhao C.-X. (2022). Drinking Water Supplemented with Acidifiers Improves the Growth Performance of Weaned Pigs and Potentially Regulates Antioxidant Capacity, Immunity, and Gastrointestinal Microbiota Diversity. Antioxidants.

