# Supplementary material for: A Commercial Clay-Based Material as a Carrier for Targeted Lysozyme Delivery in Animal Feed
Source: Nanomaterials (Basel). 2023 Nov 17;13(22):2965. doi: 10.3390/nano13222965 (PMC10674955; doi:10.3390/nano13222965)
Supplement: Supplementary file 1 [file nanomaterials-13-02965-s001.zip › nanomaterials-2681294-supplementary.pdf]

SUPPLEMENTARY MATERIALS

A Commercial Clay-Based Material as a Carrier for Targeted Lysozyme Delivery in Animal Feed

Marianna Guagliano, Cinzia Cristiani, Matteo Dell'Anno, Giovanni Dotelli, Elisabetta Finocchio, Maria Lacalamita, Ernesto Mesto, Serena Reggi, Luciana Rossi, Emanuela Schingaro

**SAMPLES**	
Abbreviation	*Full name*
LY	Lysozyme
AD	Adsorbo®
LY-AD	Organoclay composed of Adsorbo® and Lysozyme
LY(6)- AD	Organoclay loaded with 6 mg of LY per g of AD
LY(21)- AD	Organoclay loaded with 21 mg of LY per g of AD
LY(32)- AD	Organoclay loaded with 32 mg of LY per g of AD
LY(33)- AD	Organoclay loaded with 33 mg of LY per g of AD
**CHARACTERIZATION TECHNIQUES**	
Abbreviation	*Full name*
XRPD	X-Ray Powder Diffraction
TG-DTG	Thermogravimetric and Differential and Thermogravimetric analyses
FT-IR	Fourier Transform-InfraRed
ZPC	Zero Point Charge
SEM-EDX	Scanning Electron Microscopy and Energy Dispersion X-ray spectroscopy
**REACTANTS**	
Abbreviation	*Full name*
TRIS-HCl	Tris(hydroxymethyl)aminomethane hydrochloric acid
IgG-HRP	Anti-mouse immunoglobulin G horseradish peroxidase-conjugated
ABTS	2,2'-azino-bis (3-ethylbenzothiazoline-6-sulfonic acid

Table S1. Explanation of the correspondence between abbreviation and full names of: samples, characterization techniques, and the most complex reagents.

	AD	LY(6)-AD	LY(21)-AD	LY(32)-AD	LY(75)-AD
Temperature range (°C)	Weight loss (%)				
30-100	4.51	2.64	2.96	4.12	3.02
100-200	3.93	4.17	4.07	3.9	3.9
200-550	2.44	3.83	4.44	4.34	3.83
550-700	2.56	2.72	2.41	2.66	2.92

Table S2. Weigh loss of each step of the thermal decompositions of the hybrid materials with different LY content.

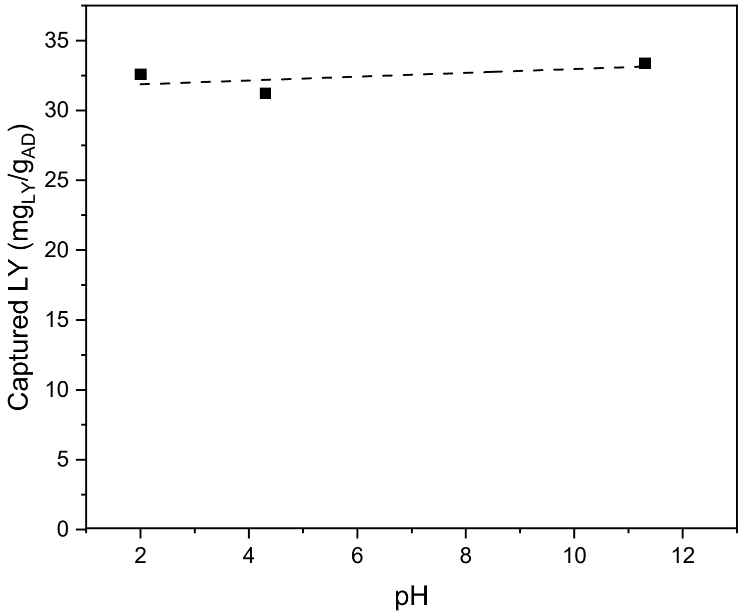

Figure S1: Captured LY as function of pH (2-11.3)

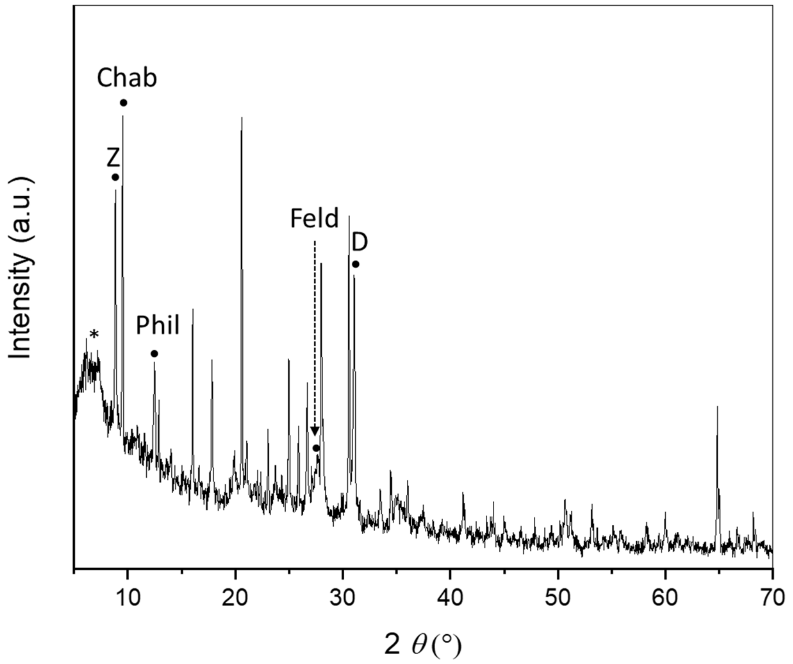

Figure S2. X-Ray Powder Diffraction Pattern of pristine Adsorbo® (AD). (Symbols: Chab = chabazite; Phil: phillipsite; Z = zeolite, Feld = feldspar; D: dolomite; *see text)

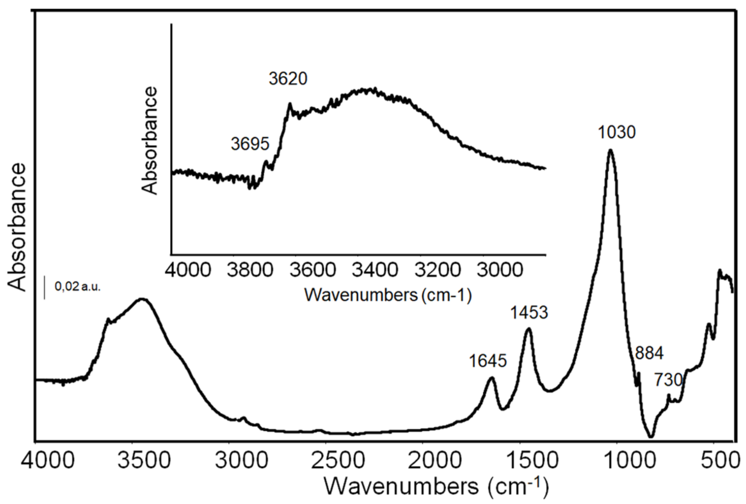

Figure S3. FT IR skeletal spectrum of Adsorbo® in KBr. Inset: FT-IR spectrum of OH stretching region, spectrum recorded in ATR mode.

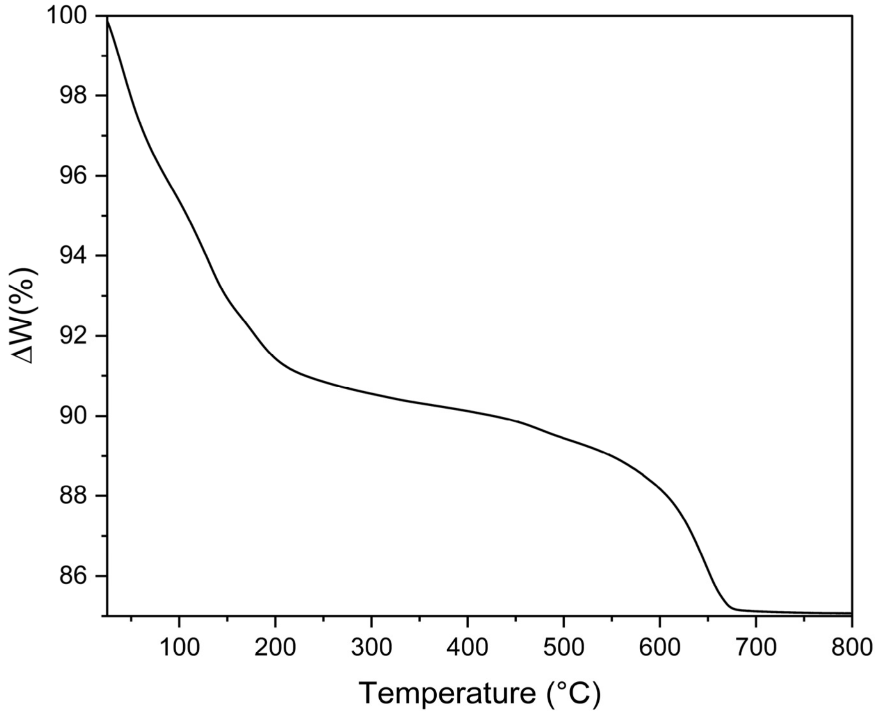

Figure S4: TG analysis of pristine carrier: Adsorbo® (AD)

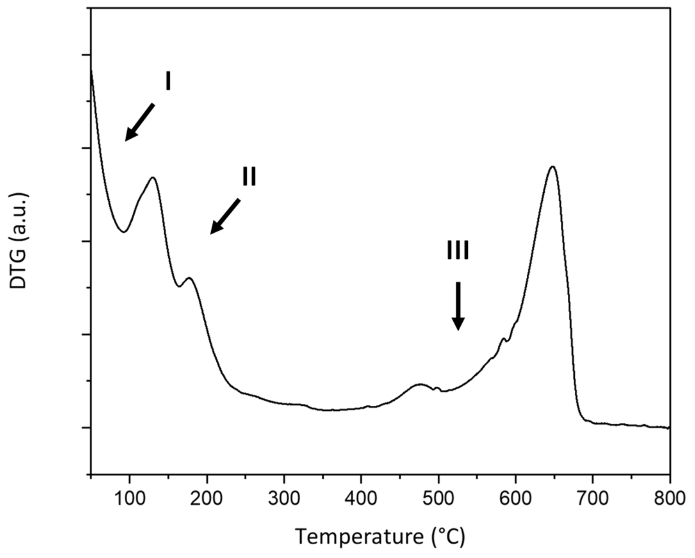

Figure S5. DTG curve of pristine Adsorbo®.

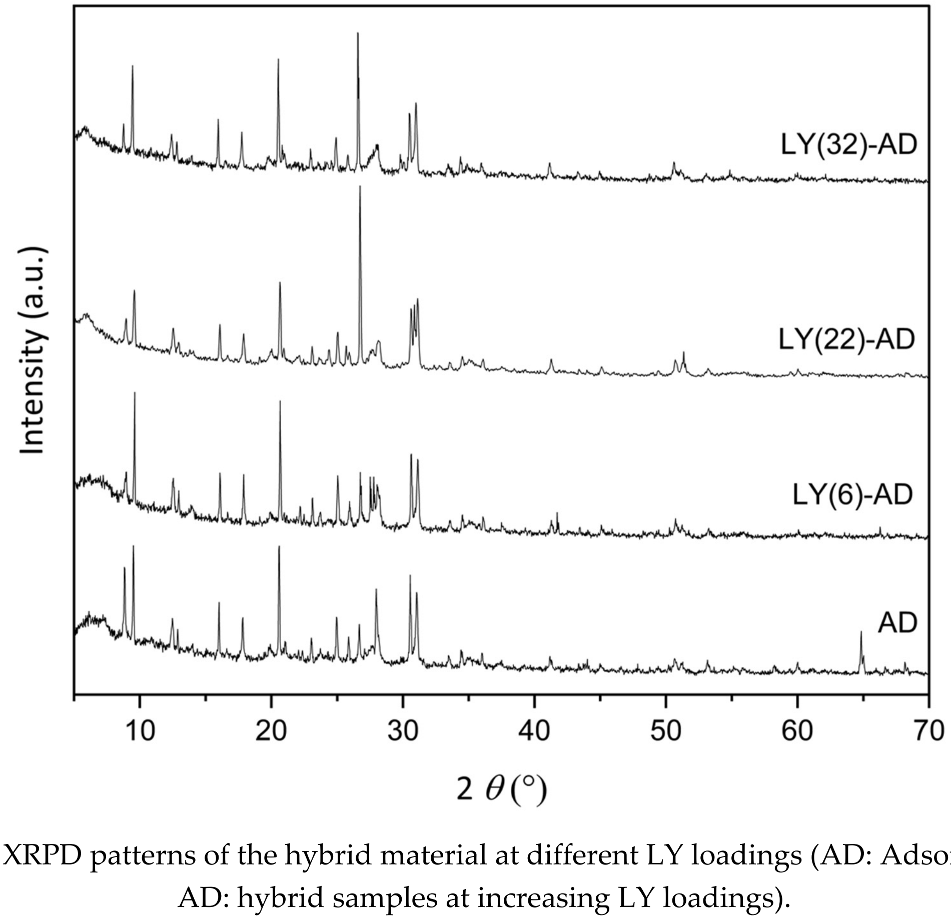

Figure S6. XRPD patterns of the hybrid material at different LY loadings (AD: Adsorbo®, LY(X)-AD: hybrid samples at increasing LY loadings).

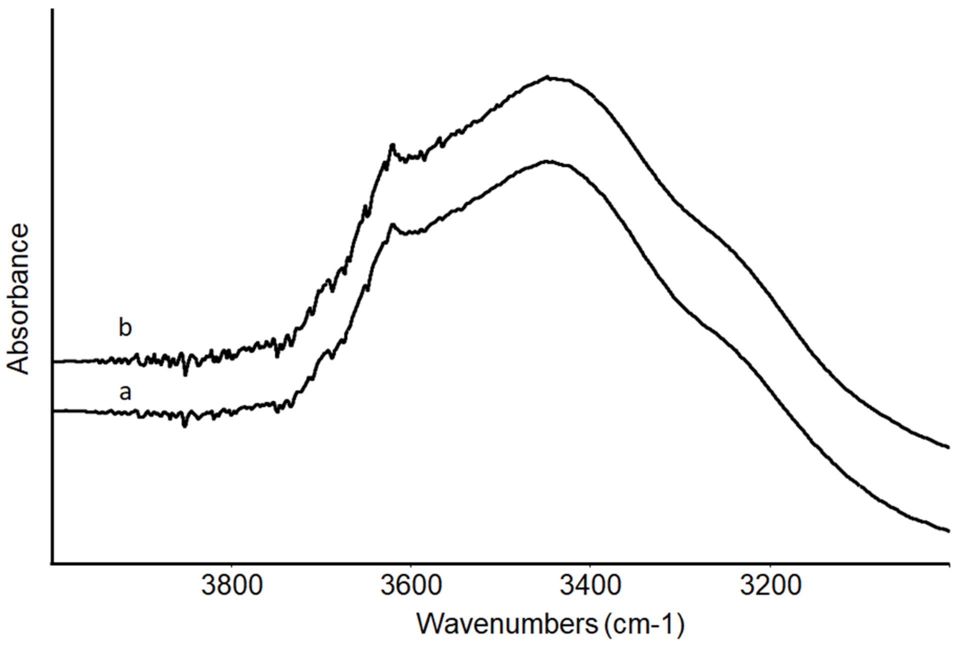

Figure S7. FT-IR spectra of: a) pristine AD, and b) LY (32)-AD.

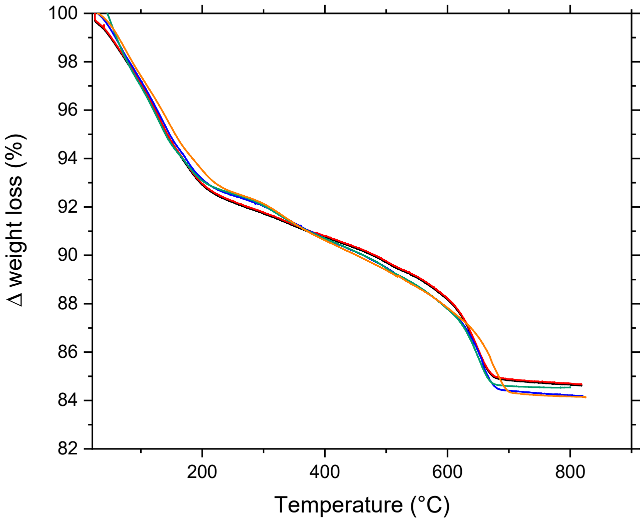

Figure S8. Unshifted curves of TG of samples at increasing LY content
